# SARS-CoV-2 B.1.617 mutations L452 and E484Q are not synergistic for antibody evasion

**DOI:** 10.1093/infdis/jiab368

**Published:** 2021-07-14

**Authors:** Isabella Ferreira, Steven Kemp, Rawlings Datir, Akatsuki Saito, Bo Meng, Partha Rakshit, Akifumi Takaori-Kondo, Yusuke Kosugi, Keiya Uriu, Izumi Kimura, Kotaro Shirakawa, Adam Abdullahi, The CITIID-NIHR BioResource COVID-19 Collaboration, The Indian SARS-CoV-2 Genomics Consortium (INSACOG), Anurag Agarwal, Seiya Ozono, Kenzo Tokunaga, The Genotype to Phenotype Japan (G2P-Japan) Consortium, Kei Sato, Ravindra K Gupta

**Affiliations:** 1 Cambridge Institute of Therapeutic Immunology & Infectious Disease (CITIID), Cambridge, UK; 2 Department of Medicine, University of Cambridge, Cambridge, UK; 3 Department of Veterinary Medicine, Faculty of Agriculture, Graduate School of Medicine and Veterinary Medicine, Center for Animal Disease Control, University of Miyazaki, Miyazaki, Japan; 4 National Centre for Disease Control, Delhi, India; 5 Department of Hematology and Oncology, Kyoto University, Kyoto, Japan; 6 Division of Systems Virology, Institute of Medical Science, University of Tokyo, Japan; 7 CSIR Institute of Genomics and Integrative Biology, Delhi, India; 8 Department of Pathology, National Institute of Infectious Diseases, Tokyo, Japan; 9 CREST, Japan Science and Technology Agency, Saitama, Japan; 10 Africa Health Research Institute, Durban, South Africa

**Keywords:** SARS-CoV-2, COVID-19, B.1.617, Indian variant, antibody escape, neutralising antibodies, infectivity, spike mutation, evasion, resistance, fitness

## Abstract

The SARS-CoV-2 B.1.617 variant emerged in the Indian state of Maharashtra in late 2020. There have been fears that two key mutations seen in the receptor binding domain L452R and E484Q would have additive effects on evasion of neutralising antibodies. We report that spike bearing L452R and E484Q confers modestly reduced sensitivity to BNT162b2 mRNA vaccine-elicited antibodies following either first or second dose. The effect is similar in magnitude to the loss of sensitivity conferred by L452R or E484Q alone. These data demonstrate reduced sensitivity to vaccine elicited neutralising antibodies by L452R and E484Q but lack of synergistic loss of sensitivity.

**Background**

Global control of the SARS-CoV-2 pandemic has yet to be realised despite availability of highly effective vaccines. Emergence of new variants with multiple mutations is likely the result of chronic infections within individuals who are immune compromised[1]. These new variants with antibody escape mutations has coincided with vaccine scale up, potentially threatening their success in controlling the pandemic[2, 3].

India experienced a wave of infections in mid 2020 that was controlled by a nationwide lockdown. Since easing of restrictions, India has seen expansion in cases of COVID-19 since March 2021. The B.1.617 variant emerged in the state of Maharashtra in late 2020/early 2021 and has spread throughout India and to at least 60 countries. It was labelled initially as a 'double mutant' since two of the mutations L452R and E484Q were matched to an in-house screening database for mutations leading to probable evasion of antibodies and/or being linked to increased transmissibility.

L452R and E484Q are located in the critical receptor binding domain that interacts with ACE2[4]. L452R was observed in the 'Epsilon Variant' B.1.429 and is associated with increase in viral load and around 20% increased transmissibility [5]. It was also associated with increased ACE2 binding, increased infectivity[6] and 3-6 fold loss of neutralisation sensitivity to vaccine elicited sera in experiments with pseudotyped virus (PV) particles[6, 7]. Little is known about E484Q, though E484K is a defining feature of two VOCs, B.1.351 and P.1, and is found alongside K417N/T as well as N501Y in these VOC. E484K has also emerged in the background of B.1.1.7[8].

**Methods**

*Phylogenetic Analysis*

All sequences excluding low-quality sequences (>5% N regions) with the L452R mutation were downloaded from https://gisaid.org on the 4th May 2021 and manually aligned to reference strain MN908947.3 with mafft v4.475 using the --keeplength --addfragments option. Sequences were de-duplicated using bbtools dedupe.sh. A random subset of 400 global sequences (excluding USA), and 100 USA sequences were then selected with seqtk and concatenated. Sequence lineages were assigned to all sequences with pangolin v2.4 (https://github.com/cov-lineages/pangolin) and pangolearn (04/05/2021).

Phylogenies were then inferred using maximum-likelihood in IQTREE v2.1.3 [9] using a GTR+R6 model and the -fast option. Mutations of interest were determined using a local instance of nextclade-cli v0.14.2 (https://github.com/nextstrain/nextclade). The inferred phylogeny was annotated in R v4.04 using ggtree v2.2.4 and rooted on the SARS-CoV-2 reference sequence, and nodes arranged in descending order. Major lineages were annotated on the phylogeny, as well as a heatmap indicating which mutations of interest were carried by each viral sequence.

*Structural Analyses*

The PyMOL Molecular Graphics System v.2.4.0 (https://github.com/schrodinger/pymol-open-source/releases) was used to map the location of the two RDB mutants L452R and E484Q onto two previously published SARS-CoV-2 spike glycoprotein structures. The two structures included a closed-conformation spike protein - PDB: 6ZGE and a spike protein in open conformation, bound to nAb H4 [10].

*Serum samples and ethical approval*

Ethical approval for use of serum samples. Controls with COVID-19 were enrolled to the NIHR BioResource Centre Cambridge under ethics review board (17/EE/0025). Protocols involving human subjects recruited at Kyoto University, Japan, were reviewed and approved by (approval numbers G0697 and G1309). All human subjects provided written informed consent.

*Cells*

HEK 293T CRL-3216, Vero CCL-81 were purchased from ATCC and maintained in Dulbecco's Modified Eagle Medium (DMEM) supplemented with 10% fetal calf serum (FCS), 100 U/ml penicillin, and 100mg/ml streptomycin. All cells were regularly tested and are mycoplasma free.

*Pseudotype virus preparation*

Plasmids encoding the spike protein of SARS-CoV-2 with a C terminal 19 amino acid deletion with D614G, were used. Mutations were introduced using Quickchange Lightning Site-Directed Mutagenesis kit (Agilent) following the manufacturer's instructions. Viral vectors were prepared by transfection of 293T cells by using Fugene HD transfection reagent (Promega). 293T cells were transfected with a mixture of 11ul of Fugene HD, 1µg of pCDNAΔ19 spike-HA, 1ug of p8.91 HIV-1 gag-pol expression vector and 1.5µg of pCSFLW (expressing the firefly luciferase reporter gene with the HIV-1 packaging signal). Viral supernatant was collected at 48 and 72h after transfection, filtered through 0.45um filter and stored at -80˚C. Infectivity was measured by luciferase detection in target 293T cells transfected with TMPRSS2 and ACE2.

*Standardisation of virus input by SYBR Green-based product-enhanced PCR assay (SG-PERT)*

The reverse transcriptase activity of virus preparations was determined by qPCR using a SYBR Green-based product-enhanced PCR assay (SG-PERT) as previously described[11]. Briefly, 10-fold dilutions of virus supernatant were lysed in a 1:1 ratio in a 2x lysis solution (made up of 40% glycerol v/v 0.25% Trition X-100 v/v 100mM KCl, RNase inhibitor 0.8 U/ml, TrisHCL 100mM, buffered to pH7.4) for 10 minutes at room temperature.

*Serum pseudotype neutralisation assay for Pfizer BNT162b2 dose 1 experiments*

Virus neutralisation assays were performed on 293T cell transiently transfected with ACE2 and TMPRSS2 using SARS-CoV-2 spike pseudotyped virus expressing luciferase[12]. Pseudotyped virus was incubated with serial dilution of heat inactivated human serum samples or convalescent plasma in duplicate for 1h at 37˚C. Virus and cell only controls were also included. Then, freshly trypsinized 293T ACE2/TMPRSS2 expressing cells were added to each well. Following 48h incubation in a 5% $CO_2$ environment at 37°C, the luminescence was measured using Steady-Glo Luciferase assay system (Promega). IC50 was calculated in GraphPad Prism v8.0.

*Establishment of stable cells for Pfizer dose 2 experiments*

The ACE2-expressing lentiviral plasmid pWPI-ACE2-zeo was generated by replacing the original EGFP gene of the lentiviral transfer plasmid pWPI (Pham et al., 2004), with the zeocin-resistant gene, and by inserting the ACE2 gene into the region immediately upstream of the internal ribosome entry site. Similarly, the TMPRSS2-expressing lentiviral plasmid pWPI-TMPRSS2-neo was created by inserting the neomycin-resistant gene and the TMPRSS2 gene into pWPI.

293T cells (4.4 x $10^5$) were cotransfected with 0.1 μg of pC-VSVg, 0.95 μg of psPAX2-IN/HiBiT (Ozono et al., 2021), and 0.95 μg of either pWPI-ACE2-zeo or pWPI-TMPRSS2-neo, using FuGENE 6 (Promega). Sixteen hours later, the cells were washed with phosphate-buffered saline, and 1 ml of fresh complete medium was added. After 24 h, the supernatants were harvested and treated with DNase I (Roche) at 37 °C for 30min. The lentivirus levels in viral supernatants were measured by the HiBiT assay, as previously described (Ozono et al., 2020). HOS cells (1 x $10^5$) were then transduced with the ACE2-expressing lentiviral vector and the TMPRSS2-expressing lentiviral vector at a multiplicity of infection of 2. After 48 h, transduced cells were maintained for zeocin (50 μg $ml^{-1}$; Thermo Ficher) and G418 (400 μg $ml^{-1}$; Nacalai) selections for 14 d.

**Results**

We subsampled SARS-CoV-2 sequences containing L452R from GISAID, and inferred a maximum likelihood phylogenetic tree (Figure 1A). We annotated the sequences based on the accompanying mutations and observed three lineages within B.1.617. B.1.617.1 has three key spike mutations L452R, E484Q and P681R, whereas B.1.617.2 is characterised by L452R, T478K and P681R (cleavage site region). There was likely loss of E484Q in B.1.617.2 given that B.1.617.3 also bears E484Q (Figure 1A), indicating E484Q was present in the ancestral virus. There are multiple other mutations in the NTD and S2 regions of B.1.617 lineages. The number of sequenced isolates of B.1.617.1 and B.1.617.2 has been steadily increasing in India (Figure 1B), though with the caveat of very low sequencing of prevalent cases and heterogeneous sampling across the country.

Spike mutations L452R and E484Q are in the receptor binding domain that not only binds ACE2[13], but is a target for neutralising antibodies[14, 15] (Figure 2A). We tested the neutralisation sensitivity of combinations of mutations found in B.1.617.1: L452R, E484Q and P681R, using a previously reported pseudotyped virus (PV) system. We tested 24 stored sera from first dose (Figure 2B) and 16 sera from second dose (Figure 2C) Pfizer BNT162b2 vaccinees against a range of spike mutation bearing PV (Figure 2B,C, Supplementary Figure 1B,C). E484Q had a similar impact on reducing neutralisation sensitivity as L452R and E484K (3.6-4.5 fold). When E484Q and L452R were combined, there was a statistically significant loss of sensitivity as compared to wild type, but the fold change of 5.1 was similar to that observed with each mutation individually with absence of evidence for an additive effect (Figure 2B, Supplementary Figure 1B). However, as expected, in some sera there was evidence for variable neutralising activity against the L452R and E484Q PVs, reflecting differential antibody responses between individuals. When we tested second dose sera (Figure 2C, Supplementary Figure 1C), similar patterns were observed between different viruses although fold changes were lower overall, likely due to increased neutralisation breadth and potency following booster vaccination[8].

Finally, with the PV system we measured spike mediated entry into target HOS cells endogenously expressing ACE2 and TMPRSS2 receptors. The E484K and L452R mutant did not have significantly higher entry efficiency compared to single mutants (Figure 2D). We also tested the entry efficiency of L452R, E484Q and P681R in a range of target cell lines, either exogenously or endogenously expressing SARS-CoV-2 receptors ACE2/TMPRSS2. The spike triple mutant exhibited similar or mildly reduced entry compared to Wuhan-1 D614G spike (Supplementary Figure 2).

**Discussion**

Here we demonstrate three lineages of B.1.617, all bearing the L452R mutation. We report key differences in amino acids between sub-lineages and focus on B.1.617.1 bearing two key RBD

mutations L452R, E484Q. *In vitro*, we find modestly reduced sensitivity of the spike protein bearing RBD mutations L452R and E484Q to BNT162b2 mRNA vaccine-elicited antibodies that is similar in magnitude to the loss of sensitivity conferred by L452R or E484Q alone. P681R did not appear to alter the sensitivity to vaccine sera or to alter the entry efficiency conferred by spike protein on lentiviral particles. These data demonstrate reduced sensitivity to vaccine elicited neutralising antibodies by the RBD bearing L452R and E484Q but lack of synergistic loss of sensitivity.

## References

1. Kemp SA, Collier DA, Datir RP et al. SARS-CoV-2 evolution during treatment of chronic infection. *Nature* 2021.
2. Gupta RK. Will SARS-CoV-2 variants of concern affect the promise of vaccines? *Nature reviews Immunology* 2021.
3. Hacisuleyman E, Hale C, Saito Y et al. Vaccine Breakthrough Infections with SARS-CoV-2 Variants. *New England Journal of Medicine* 2021.
4. Greaney AJ, Starr TN, Gilchuk P et al. Complete Mapping of Mutations to the SARS-CoV-2 Spike Receptor-Binding Domain that Escape Antibody Recognition. *Cell host & microbe* 2020.
5. Deng X, Garcia-Knight MA, Khalid MM et al. Transmission, infectivity, and antibody neutralization of an emerging SARS-CoV-2 variant in California carrying a L452R spike protein mutation. *medRxiv* 2021.
6. Motozono C, Toyoda M, Zahradnik J et al. An emerging SARS-CoV-2 mutant evading cellular immunity and increasing viral infectivity. *bioRxiv* 2021: 2021.04.02.438288.
7. McCallum M, Bassi J, Marco AD et al. SARS-CoV-2 immune evasion by variant B.1.427/B.1.429. *bioRxiv* 2021: 2021.03.31.437925.
8. Collier DA, De Marco A, Ferreira I et al. Sensitivity of SARS-CoV-2 B.1.1.7 to mRNA vaccine-elicited antibodies. *Nature* 2021.
9. Minh BQ, Schmidt H, Chernomor O et al. IQ-TREE 2: New models and efficient methods for phylogenetic inference in the genomic era. *bioRxiv* 2019: 849372.
10. Rapp M, Guo Y, Reddem ER et al. Modular basis for potent SARS-CoV-2 neutralization by a prevalent VH1-2-derived antibody class. *Cell Reports* 2021; **35**: 108950.
11. Vermeire J, Naessens E, Vanderstraeten H et al. Quantification of reverse transcriptase activity by real-time PCR as a fast and accurate method for titration of HIV, lenti- and retroviral vectors. *PloS one* 2012; **7**: e50859-e.
12. Mlcochova P, Collier D, Ritchie A et al. Combined point of care nucleic acid and antibody testing for SARS-CoV-2 following emergence of D614G Spike Variant. *Cell Rep Med* 2020: 100099.
13. Starr TN, Greaney AJ, Hilton SK et al. Deep Mutational Scanning of SARS-CoV-2 Receptor Binding Domain Reveals Constraints on Folding and ACE2 Binding. *Cell* 2020; **182**: 1295-310 e20.
14. Barnes CO, Jette CA, Abernathy ME et al. SARS-CoV-2 neutralizing antibody structures inform therapeutic strategies. *Nature* 2020; **588**: 682-7.
15. Barnes CO, West AP, Jr., Huey-Tubman KE et al. Structures of Human Antibodies Bound to SARS-CoV-2 Spike Reveal Common Epitopes and Recurrent Features of Antibodies. *Cell* 2020; **182**: 828-42 e16.

**Acknowledgments**

We would like to thank the Department of Biotechnology, NCDC, RKG is supported by a Wellcome Trust Senior Fellowship in Clinical Science (WT108082AIA). KS is supported by AMED Research Program on Emerging and Re-emerging Infectious Diseases (20fk0108413). We would like to thank COG-UK. COG-UK is supported by funding from the Medical Research Council (MRC) part of UK Research & Innovation (UKRI), the National Institute of Health Research (NIHR) and Genome Research Limited, operating as the Wellcome Sanger Institute. This study was supported by the Cambridge NIHRB Biomedical Research Centre. SAK is supported by the Bill and Melinda Gates Foundation via PANGEA grant: OPP1175094. We would like to thank Paul Lehner. We thank Wendy Barclay and Thomas Peacock for helpful discussions and the Geno2pheno UK consortium. We thank Izumi Kimura, Keiya Uriu and Yusuke Kosugi for technical supports. This study was also partly supported by Rosetrees Trust.

## Figure Legends

**Figure 1. SARS-CoV-2 B.1.617 variant emerging in India A**. Maximum-likelihood phylogeny of lineages bearing L452R in spike. All sequences with the L452R mutation were downloaded from https://gisaid.org and manually aligned to reference strain MN908947.3 with mafft. Sequences were de-duplicated and a random subset of 400 global sequences, and 100 USA sequences were then selected with seqtk. All sequence lineages were assigned using pangolin v2.4. Major lineages are indicated as straight lines adjacent to the heatmap, alongside mutations of current interest. The phylogeny was inferred with IQTREE2 v2.1.3. **B.** The number of B.1617 cases per month in India in the first half of 2021.

**Figure 2. Entry efficiency and neutralisation sensitivity of B.1.617 mutant pseudotyped viruses following mRNA vaccination** A. Surface representation of the spike protein in open formation with neutralising antibody H4 (pink spheres, PDB: 7L58, Rapp et al, 2021) bound to one monomer of the spike protein. Residues L452 and E484 are indicated with red and green sphere, respectively. Ribbon representation of the interaction between the neutralising antibody H4 and the RBD of a spike monomer. Neutralisation by **B. first dose** and **C. second dose** mRNA vaccine-elicited sera against wild type and mutant SARS-CoV-2 spike pseudotyped viruses. Reciprocal geometric mean titre (GMT) shown with 95% CI. *$p<0.05$, ** $p<0.01$, ***$p<0.001$, ****$p<0.0001$. **D.** Virus infectivity of pseudotyped virus (PV) bearing indicated spike mutations. PV were generated in 293T cells and used to infect HOS cells transduced with ACE2 and TMPRSS2. Input virus was normalized for protein expression. Data are technical triplicates and mean with SE is plotted. Data are representative of two independent experiments.

**Supplementary Figure 1: Neutralisation sensitivity of B.1.617 mutant pseudotyped viruses following mRNA vaccination A**. Representative neutralisation curves from mRNA first dose vaccine sera tested against PV bearing spike mutations observed in B.1.617.1 RBD. Error bars represent standard error of mean of technical replicates. Data are representative of two independent experiments. Neutralisation by **B. first dose** and **C. second dose** mRNA vaccine-elicited sera against wild type and mutant SARS-CoV-2 spike pseudotyped viruses. Reciprocal geometric Mean Titre (GMT) shown *p<0.05, ** p<0.01, ***p<0.001, ****p<0.0001. **D.** Reciprocal geometric mean titre (GMT) for first and second dose sera against SARS-CoV-2 spike PV with standard error of the mean.

**Supplementary Figure 2: Entry efficiency of B.1.617 mutant pseudotyped viruses.** Virus infectivity of pseudotyped virus (PV) bearing indicated spike mutations. PV were generated in 293T cells, filtered and then used to infect a range of target cells. Luciferase was measured 48 hours after infection. Input virus inoculum was corrected for genome copy input using SG-PERT. Mean is plotted with error bars representing SEM.  Data are representative of two experiments.

Figure 1

A
B
delta69/70
delta144
deltaF157/R158
T478K
E484Q
N501Y
P681R
L452R
A.27
A.2.5
B.1.36.35
B.1362
B.1.427/
B.1.429
L.3
C.36
C.16
B.1.617.3
B.1.617.2
B.1.617.1
0.0003
B.1.617.1
B.1.617.2
B.1.617.3
Number of Cases
2000
1500
1000
500
0
Mar-21
Apr-21
May-21
Jun-21
Date (MM-YY)
Country
Africa
Asia
Europe
India
North America
Oceania
South America
United Kingdom

Figure 2

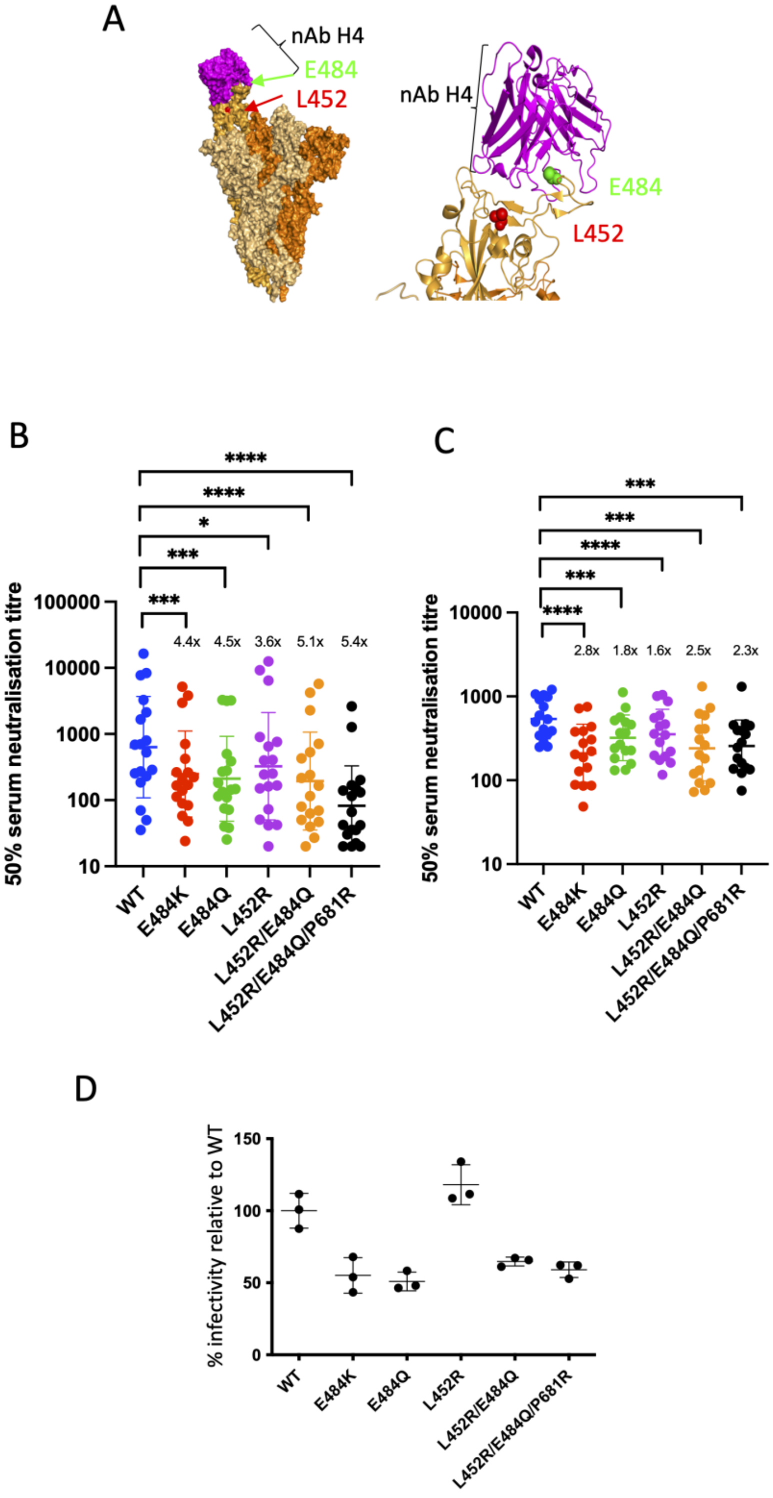

A
nAb H4
E484
L452
nAb H4
E484
L452
B
50% serum neutralisation titre
100000
10000
1000
100
10
****
****
*
***
***
4.4x
4.5x
3.6x
5.1x
5.4x
WT
E484K
E484Q
L452R
L452R/E484Q
L452R/E484Q/P681R
C
50% serum neutralisation titre
10000
1000
100
10
***
***
****
***
****
2.8x
1.8x
1.6x
2.5x
2.3x
WT
E484K
E484Q
L452R
L452R/E484Q
L452R/E484Q/P681R
D
% infectivity relative to WT
150
100
50
0
WT
E484K
E484Q
L452R
L452R/E484Q
L452R/E484Q/P681R

## Supplementary Material

jiab368_suppl_Supplementary_Materials

