# SARS-CoV-2 B.1.617 Mutations L452R and E484Q Are Not Synergistic for Antibody Evasion

**DOI:** 10.1093/infdis/jiab368

**Published:** 2021-09-09

**Authors:** Isabella A T M Ferreira, Steven A Kemp, Rawlings Datir, Akatsuki Saito, Bo Meng, Partha Rakshit, Akifumi Takaori-Kondo, Yusuke Kosugi, Keiya Uriu, Izumi Kimura, Kotaro Shirakawa, Adam Abdullahi, Anurag Agarwal, Seiya Ozono, Kenzo Tokunaga, Kei Sato, Ravindra K Gupta, CITIID-NIHR BioResource COVID-19 Collaboration, Indian SARS-CoV-2 Genomics Consortium, The Genotype to Phenotype Japan (G2P-Japan) Consortium

**Affiliations:** 1 Cambridge Institute of Therapeutic Immunology and Infectious Disease, Cambridge,United Kingdom; 2 Department of Medicine, University of Cambridge, Cambridge,United Kingdom; 3 Department of Veterinary Medicine, Faculty of Agriculture, University of Miyazaki, Miyazaki,Japan; 4 National Centre for Disease Control, Delhi,India; 5 Department of Hematology and Oncology, Kyoto University, Kyoto,Japan; 6 Divisionof Systems Virology, Institute of Medical Science, University of Tokyo, Tokyo, Japan; 7 CSIR Institute of Genomics and Integrative Biology, Delhi,India; 8 Department of Pathology, National Institute of Infectious Diseases, Tokyo,Japan; 9 CREST, Japan Science and Technology Agency, Saitama,Japan; 10 Africa Health Research Institute, Durban,South Africa

**Keywords:** antibody escape, B.1.617, COVID-19, evasion, fitness, Indian variant, infectivity, neutralizing antibodies, resistance, SARS-CoV-2, spike mutation

## Abstract

The SARS-CoV-2 B.1.617 variant emerged in the Indian state of Maharashtra in late 2020. There have been fears that 2 key mutations seen in the receptor-binding domain, L452R and E484Q, would have additive effects on evasion of neutralizing antibodies. We report that spike bearing L452R and E484Q confers modestly reduced sensitivity to BNT162b2 mRNA vaccine-elicited antibodies following either first or second dose. The effect is similar in magnitude to the loss of sensitivity conferred by L452R or E484Q alone. These data demonstrate reduced sensitivity to vaccine-elicited neutralizing antibodies by L452R and E484Q but lack of synergistic loss of sensitivity.

Global control of the severe acute respiratory syndrome coronavirus 2 (SARS-CoV-2) pandemic has yet to be realized despite availability of highly effective vaccines. Emergence of new variants with multiple mutations is likely the result of chronic infections within individuals who are immune compromised [1]. These new variants with antibody escape mutations have coincided with vaccine scale up, potentially threatening their success in controlling the pandemic.

India experienced a wave of infections in mid-2020 that was controlled by a nationwide lockdown. After easing of restrictions, India has seen expansion in cases of coronavirus disease 2019 (COVID-19) since March 2021. The B.1.617 variant emerged in the state of Maharashtra in late 2020/early 2021 and has spread throughout India and to at least 60 countries. It was labelled initially as a double mutant because 2 of the mutations, L452R and E484Q, were matched to an in-house screening database for mutations leading to probable evasion of antibodies and/or being linked to increased transmissibility.

L452R and E484Q are located in the critical receptor-binding domain (RBD) that interacts with angiotensin-converting enzyme 2 (ACE2) [2]. L452R was observed in the epsilon variant B.1.429 and is associated with increase in viral load and around 20% increased transmissibility [3]. It was also associated with increased ACE2 binding, increased infectivity [4], and 3- to 6-fold loss of neutralization sensitivity to vaccine-elicited sera in experiments with pseudotyped virus (PV) particles [5]. Little is known about E484Q, although E484K is a defining feature of 2 variants of concern (VOCs), B.1.351 and P.1, and is found alongside K417N/T as well as N501Y in these VOC. E484K has also emerged in the background of B.1.1.7 [6].

## METHODS

### Phylogenetic Analysis

All sequences excluding low-quality sequences (>5% N regions) with the L452R mutation were downloaded from GISAID database (https://gisaid.org) on 4 May 2021 and manually aligned to reference strain MN908947.3 with mafft v4.475 using the --keeplength --addfragments option. Sequences were deduplicated using bbtools dedupe.sh. A random subset of 400 global sequences (excluding US sequences) and 100 US sequences were then selected with seqtk and concatenated. Sequence lineages were assigned to all sequences with pangolin version 2.4 (https://github.com/cov-lineages/pangolin) and pangolearn (4 May 2021).

Phylogenies were then inferred using maximum-likelihood in IQTREE version 2.1.3 [7] using a GTR + R6 model and the -fast option. Mutations of interest were determined using a local instance of nextclade-cli version 0.14.2 (https://github.com/nextstrain/nextclade). The inferred phylogeny was annotated in R version 4.04 using ggtree version 2.2.4 and rooted on the SARS-CoV-2 reference sequence, and nodes arranged in descending order. Major lineages were annotated on the phylogeny, as well as a heatmap indicating which mutations of interest were carried by each viral sequence.

### Structural Analyses

The PyMOL Molecular Graphics System version 2.4.0 (https://github.com/schrodinger/pymol-open-source/releases) was used to map the location of the 2 RBD mutants L452R and E484Q onto 2 previously published SARS-CoV-2 spike glycoprotein structures. The 2 structures included a closed-conformation spike protein, Protein Data Bank 6ZGE and a spike protein in open conformation, bound to nAb H4 [8].

### Serum Samples and Ethical Approval

Ethical approval was sought for use of serum samples. Controls with COVID-19 were enrolled to the National Institute of Health Research BioResource Centre Cambridge under ethics review board (17/EE/0025). Protocols involving human subjects recruited at Kyoto University, Japan, were reviewed and approved by (approval numbers G0697 and G1309). All human subjects provided written informed consent.

### Cells

HEK 293T CRL-3216, Vero CCL-81 were purchased from American Type Culture Collection and maintained in Dulbecco’s Modified Eagle Medium (DMEM) supplemented with 10% fetal calf serum, 100 U/mL penicillin, and 100 mg/mL streptomycin. All cells were regularly tested and were mycoplasma free.

### Pseudotype Virus Preparation

Plasmids encoding the spike protein of SARS-CoV-2 with a C terminal 19 amino acid deletion with D614G were used. Mutations were introduced using Quickchange Lightning Site-Directed Mutagenesis kit (Agilent) following the manufacturer’s instructions. Viral vectors were prepared by transfection of 293T cells by using Fugene HD transfection reagent (Promega). 293T cells were transfected with a mixture of 11 µL of Fugene HD, 1 µg of pCDNAΔ19 spike-HA, 1 µg of p8.91 HIV-1 gag-pol expression vector, and 1.5 µg of pCSFLW (expressing the firefly luciferase reporter gene with the HIV-1 packaging signal). Viral supernatant was collected at 48 and 72 hours after transfection, filtered through a 0.45-µm filter and stored at −80°C. Infectivity was measured by luciferase detection in target 293T cells transfected with TMPRSS2 and ACE2.

### Standardization of Virus Input by SYBR Green-Based Product-Enhanced PCR ASSAY

The reverse transcriptase activity of virus preparations was determined by quantitative polymerase chain reaction (qPCR) using a SYBR Green-based product-enhanced PCR assay (SG-PERT) as previously described [9]. Briefly, 10-fold dilutions of virus supernatant were lysed in a 1:1 ratio in a 2× lysis solution (40% glycerol v/v, 0.25% Trition X-100 v/v, 100 mM KCl, RNase inhibitor 0.8 U/mL, TrisHCL 100mM, buffered to pH 7.4) for 10 minutes at room temperature.

### Serum Pseudotype Neutralization Assay for Pfizer BNT162b2 Dose 1 Experiments

Virus neutralization assays were performed on 293T cells transiently transfected with ACE2 and TMPRSS2 using SARS-CoV-2 spike PV expressing luciferase [10]. PV was incubated with serial dilution of heat inactivated human serum samples in duplicate for 1h at 37°C. Virus and cell-only controls were also included. Then, freshly trypsinized 293T ACE2/TMPRSS2-expressing cells were added to each well. Following 48-hour incubation in a 5% CO_2_ environment at 37°C, the luminescence was measured using Steady-Glo Luciferase assay system (Promega). Half maximum inhibitory concentration (IC_50_) was calculated in GraphPad Prism version 8.0.

### Establishment of Stable Cells for Pfizer Dose 2 Experiments

The ACE2-expressing lentiviral plasmid pWPI-ACE2-zeo was generated by replacing the original *EGFP* gene of the lentiviral transfer plasmid pWPI [11], with the zeocin-resistant gene, and by inserting the *ACE2* gene into the region immediately upstream of the internal ribosome entry site. Similarly, the TMPRSS2-expressing lentiviral plasmid pWPI-TMPRSS2-neo was created by inserting the neomycin-resistant gene and the *TMPRSS2* gene into pWPI.

293T cells (4.4 × 10^5^) were cotransfected with 0.1 μg of pC-VSVg, 0.95 μg of psPAX2-IN/HiBiT (Ozono et al, 2021), and 0.95 μg of either pWPI-ACE2-zeo or pWPI-TMPRSS2-neo, using FuGENE 6 (Promega). Sixteen hours later, the cells were washed with phosphate-buffered saline and 1 mL of fresh complete medium was added. After 24 hours, the supernatants were harvested and treated with DNase I (Roche) at 37°C for 30 minutes. The lentivirus levels in viral supernatants were measured by the HiBiT assay, as previously described (Ozono et al, 2020). HOS cells (1 × 10^5^) were then transduced with the ACE2-expressing lentiviral vector and the TMPRSS2-expressing lentiviral vector at a multiplicity of infection of 2. After 48 hours, transduced cells were maintained for zeocin (50 μg/mL; Thermo Fisher) and G418 (400 μg/mL; Nacalai) selections for 14 days.

## RESULTS

**Figure 1.**
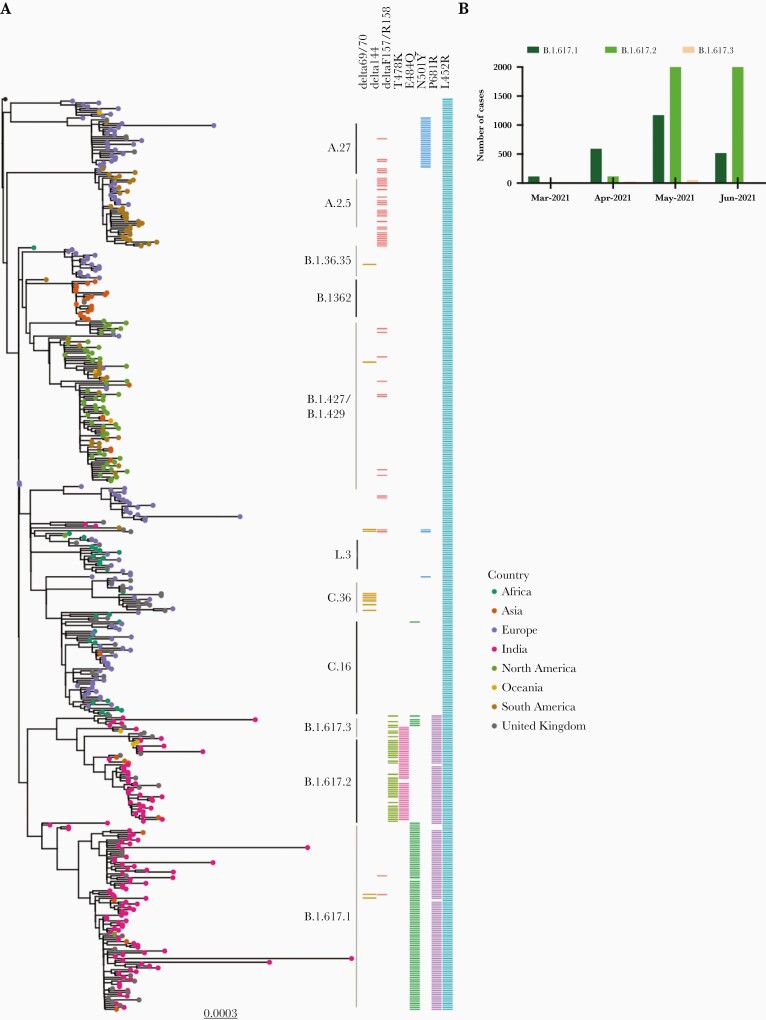
Severe acute respiratory syndrome coronavirus 2 (SARS-CoV-2) B.1.617 variant emerging in India *A*, Maximum-likelihood phylogeny of lineages bearing L452R in spike. All sequences with the L452R mutation were downloaded from https://gisaid.org and manually aligned to reference strain MN908947.3 with mafft. Sequences were deduplicated and a random subset of 400 global sequences and 100 US sequences were then selected with seqtk. All sequence lineages were assigned using pangolin version 2.4. Major lineages are indicated as straight lines adjacent to the heatmap, alongside mutations of current interest. The phylogeny was inferred with IQTREE2 version 2.1.3. *B*, The number of B.1617 cases per month in India in the first half of 2021.

We subsampled SARS-CoV-2 sequences containing L452R from GISAID database, and inferred a maximum likelihood phylogenetic tree (Figure 1A). We annotated the sequences based on the accompanying mutations and observed 3 lineages within B.1.617. B.1.617.1 has 3 key spike mutations, L452R, E484Q, and P681R, whereas B.1.617.2 is characterized by L452R, T478K, and P681R (cleavage site region). There was likely loss of E484Q in B.1.617.2 given that B.1.617.3 also bears E484Q (Figure 1A), indicating E484Q was present in the ancestral virus. There are multiple other mutations in the N-terminal domain and S2 regions of B.1.617 lineages. The number of sequenced isolates of B.1.617.1 and B.1.617.2 has been steadily increasing in India (Figure 1B), although with the caveat of very low sequencing of prevalent cases and heterogeneous sampling across the country.

**Figure 2.**
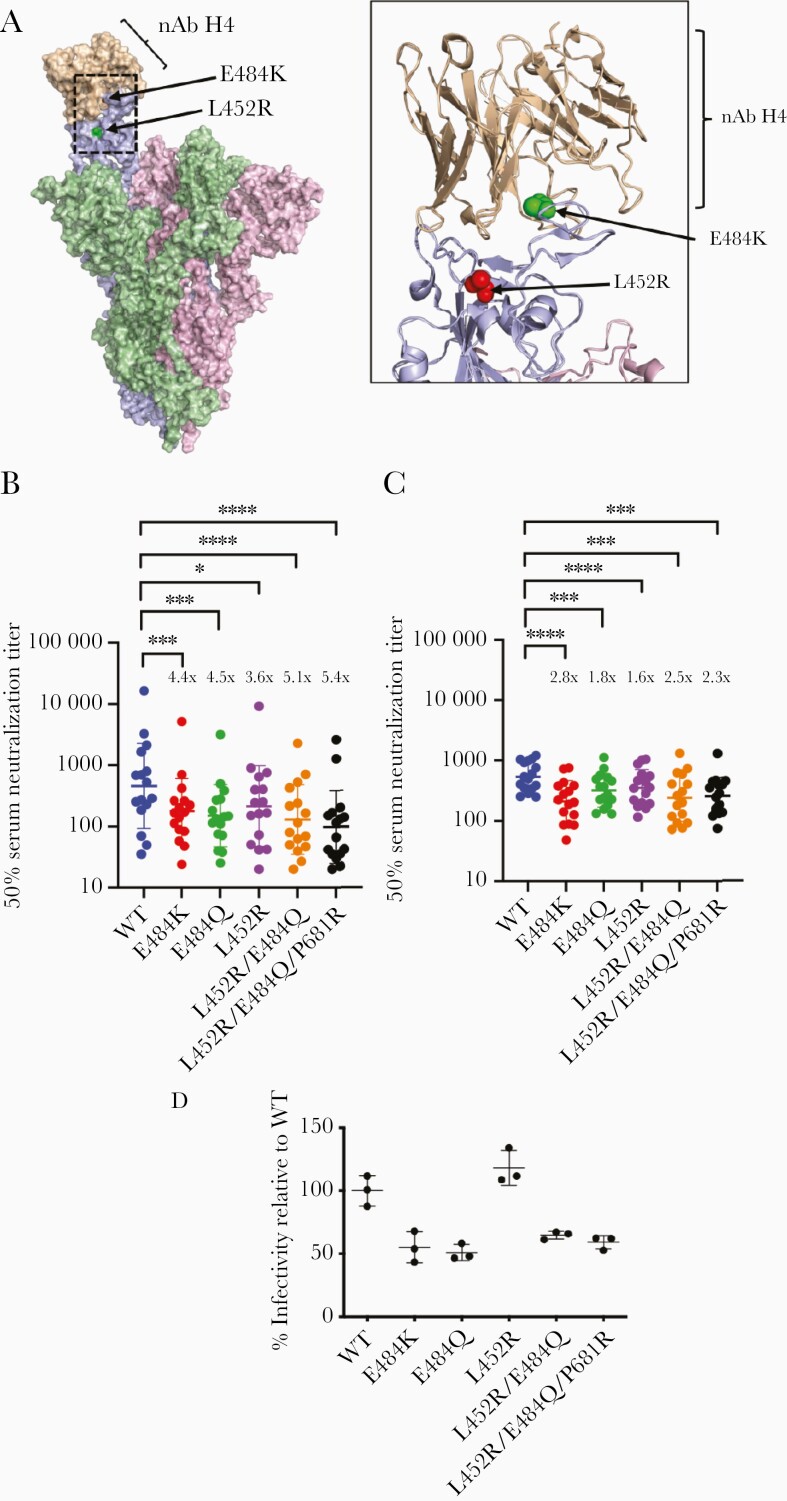
Entry efficiency and neutralization sensitivity of B.1.617 mutant pseudotyped viruses following mRNA vaccination *A*, Surface representation of the spike protein in open formation with neutralizing antibody H4 (pink spheres, Protein Data Bank 7L58, [8] ) bound to 1 monomer of the spike protein. Residues L452 and E484 are indicated with red and green spheres, respectively. Ribbon is a representation of the interaction between the neutralizing antibody H4 and the receptor-binding domain of a spike monomer. Neutralization by (*B*) first-dose and (*C*) second-dose mRNA vaccine-elicited sera against wild type (WT) and mutant severe acute respiratory syndrome coronavirus 2 (SARS-CoV-2) spike pseudotyped viruses. Reciprocal geometric mean titer shown with 95% confidence interval. *P < .05, ***P < .001, ****P < .0001. *D*, Virus infectivity of pseudotyped virus (PV) bearing indicated spike mutations. PVs were generated in 293T cells and used to infect HOS cells transduced with ACE2 and TMPRSS2. Input virus was normalized for protein expression. Data are technical triplicates and mean with SE is plotted. Data are representative of 2 independent experiments.

Spike mutations L452R and E484Q are in the RBD that not only binds ACE2 [13] but is also a target for neutralizing antibodies [14, 15] (Figure 2A). We tested the neutralization sensitivity of combinations of mutations found in B.1.617.1 (L452R, E484Q, and P681R) using a previously reported PV system. We tested 24 stored sera from first-dose (Figure 2B) and 16 sera from second-dose (Figure 2C) Pfizer BNT162b2 vaccinees against a range of spike mutation-bearing PV (Figure 2B and 2C and Supplementary Figure 1B and 1C). E484Q had a similar impact on reducing neutralization sensitivity as L452R and E484K (3.6–4.5 fold). When E484Q and L452R were combined, there was a statistically significant loss of sensitivity as compared to wild type, but the fold change of 5.1 was similar to that observed with each mutation individually with absence of evidence for an additive effect (Figure 2B and Supplementary Figure 1B). However, as expected, in some sera there was evidence for variable neutralizing activity against the L452R and E484Q PVs, reflecting differential antibody responses between individuals. When we tested second-dose sera (Figure 2C and Supplementary Figure 1C), similar patterns were observed between different viruses although fold changes were lower overall, likely due to increased neutralization breadth and potency following booster vaccination [8].

Finally, with the PV system we measured spike-mediated entry into target HOS cells endogenously expressing ACE2 and TMPRSS2 receptors. The E484K and L452R mutant did not have significantly higher entry efficiency compared to single mutants (Figure 2D). We also tested the entry efficiency of L452R, E484Q, and P681R in a range of target cell lines, either exogenously or endogenously expressing SARS-CoV-2 receptors ACE2/TMPRSS2. The spike triple mutant exhibited similar or mildly reduced entry compared to Wuhan-1 D614G spike (Supplementary Figure 2).

## Discussion

Here we demonstrate 3 lineages of B.1.617, all bearing the L452R mutation. We report key differences in amino acids between sublineages and focus on B.1.617.1 bearing 2 key RBD mutations, L452R, E484Q. In vitro, we find modestly reduced sensitivity of the spike protein bearing RBD mutations L452R and E484Q to BNT162b2 mRNA vaccine-elicited antibodies that is similar in magnitude to the loss of sensitivity conferred by L452R or E484Q alone. P681R did not appear to alter the sensitivity to vaccine sera or to alter the entry efficiency conferred by spike protein on lentiviral particles. These data demonstrate reduced sensitivity to vaccine-elicited neutralizing antibodies by the RBD bearing L452R and E484Q but lack of synergistic loss of sensitivity.

## Supplementary Data

Supplementary materials are available at *The Journal of Infectious Diseases* online. Consisting of data provided by the authors to benefit the reader, the posted materials are not copyedited and are the sole responsibility of the authors, so questions or comments should be addressed to the corresponding author.

jiab368_suppl_Supplementary_Figures

## Notes

***Acknowledgments.*** We thank the Department of Biotechnology, National Centre for Disease Control (NCDC); Paul Lehner; Wendy Barclay and Thomas Peacock for helpful discussions; the Geno2pheno UK consortium.

***Financial support.*** This work was supported by the Wellcome Trust (grant number WT108082AIA Senior Fellowship in Clinical Science to R. K. G.); Japanese Agency for Medical Research and Development (AMED) Research Program on Emerging and Re-emerging Infectious Diseases (grant number 20fk0108413 to K. S.); Cambridge NIHR Biomedical Research Centre; S. A. K. was funded by the Bill and Melinda Gates Foundation via the Phylogenetics And Networks for Generalised Epidemics in Africa (PANGEA) (grant number OPP1175094); and the Rosetrees Trust. I A. T. M. F. is funded by a Sub-Saharan African Network for TB/HIV Research Excellence (SANTHE, a Developing Excellence in Leadership, Training and Science Developing (DELTAS) Africa Initiative (grant number DEL-15– 006)) fellowship.

***Potential conflicts of interest.*** All authors: No reported conflicts of interest. All authors have submitted the ICMJE Form for Disclosure of Potential Conflicts of Interest. Conflicts that the editors consider relevant to the content of the manuscript have been disclosed.

## References

[1] Kemp SA, Collier DA, Datir RP, et al; CITIID-NIHR BioResource COVID-19 Collaboration; COVID-19 Genomics UK (COG-UK) Consortium. SARS-CoV-2 evolution during treatment of chronic infection. Nature 2021; 592:277–82. 33545711 10.1038/s41586-021-03291-y PMC7610568

[2] Greaney AJ, Starr TN, Gilchuk P, et al. Complete Mapping of Mutations to the SARS-CoV-2 Spike Receptor-Binding Domain that Escape Antibody Recognition. Cell host & microbe 2020. 10.1016/j.chom.2020.11.007 PMC7676316 33259788

[3] McCallum M, Bassi J, De Marco A, et al. SARS-CoV-2 immune evasion by the B. 1.427/B. 1.429 variant of concern. Science 2021. 10.1126/science.abi7994 PMC9835956 34210893

[4] Deng X, Garcia-Knight MA, Khalid MM, et al. Transmission, infectivity, and antibody neutralization of an emerging SARS-CoV-2 variant in California carrying a L452R spike protein mutation. medRxiv 2021.

[5] Motozono C, Toyoda M, Zahradnik J, et al. An emerging SARS-CoV-2 mutant evading cellular immunity and increasing viral infectivity. bioRxiv 2021:2021.04.02.438288.

[6] Collier DA, De Marco A, Ferreira I, et al. Sensitivity of SARS-CoV-2 B.1.1.7 to mRNA vaccine-elicited antibodies. Nature 2021. 10.1038/s41586-021-03412-7 PMC7616976 33706364

[7] Minh BQ, Schmidt HA, Chernomor O, et al. IQ-TREE 2: new models and efficient methods for phylogenetic inference in the genomic era. Mol Biol Evol 2020; 37:1530–4. 32011700 10.1093/molbev/msaa015 PMC7182206

[8] Rapp M, Guo Y, Reddem ER, et al. Modular basis for potent SARS-CoV-2 neutralization by a prevalent VH1-2-derived antibody class. Cell Reports 2021; 35:108950. 33794145 10.1016/j.celrep.2021.108950 PMC7972811

[9] Vermeire J, Naessens E, Vanderstraeten H, et al. Quantification of reverse transcriptase activity by real-time PCR as a fast and accurate method for titration of HIV, lenti- and retroviral vectors. PloS one 2012; 7:e50859-e. 23227216 10.1371/journal.pone.0050859 PMC3515444

[10] Mlcochova P, Collier D, Ritchie A, et al. Combined point of care nucleic acid and antibody testing for SARS-CoV-2 following emergence of D614G Spike Variant. Cell Rep Med 2020;100099. 32905045 10.1016/j.xcrm.2020.100099 PMC7462534

[11] Pham HM, Arganaraz ER, Groschel B, Trono D, Lama J. Lentiviral vectors interfering with virus-induced CD4 down-modulation potently block human immunodeficiency virus type 1 replication in primary lymphocytes. Journal of virology 2004; 78:13072–81. 15542659 10.1128/JVI.78.23.13072-13081.2004 PMC524995

[12] Ozono S, Zhang Y, Tobiume M, Kishigami S, Tokunaga K. Super-rapid quantitation of the production of HIV-1 harboring a luminescent peptide tag. The Journal of biological chemistry 2020; 295:13023–30. 32719008 10.1074/jbc.RA120.013887 PMC7489901

[13] Starr TN, Greaney AJ, Hilton SK, et al. Deep mutational scanning of SARS-CoV-2 receptor binding domain reveals constraints on folding and ACE2 binding. Cell 2020; 182:1295–310.e20. 32841599 10.1016/j.cell.2020.08.012 PMC7418704

[14] Barnes CO, Jette CA, Abernathy ME, et al. SARS-CoV-2 neutralizing antibody structures inform therapeutic strategies. Nature 2020; 588:682–7. 33045718 10.1038/s41586-020-2852-1 PMC8092461

[15] Barnes CO, West AP Jr, Huey-Tubman KE, et al. Structures of human antibodies bound to SARS-CoV-2 spike reveal common epitopes and recurrent features of antibodies. Cell 2020; 182:828–42.e16. 32645326 10.1016/j.cell.2020.06.025 PMC7311918

