# Supplementary material for: SARS-CoV-2 B.1.617 Mutations L452R and E484Q Are Not Synergistic for Antibody Evasion
Source: J Infect Dis. 2021 Sep 9;224(6):989–94. doi: 10.1093/infdis/jiab368 (PMC8420622; doi:10.1093/infdis/jiab368)
Supplement: jiab368_suppl_Supplementary_Figures [file jiab368_suppl_supplementary_figures.pptx]

## Slide 1
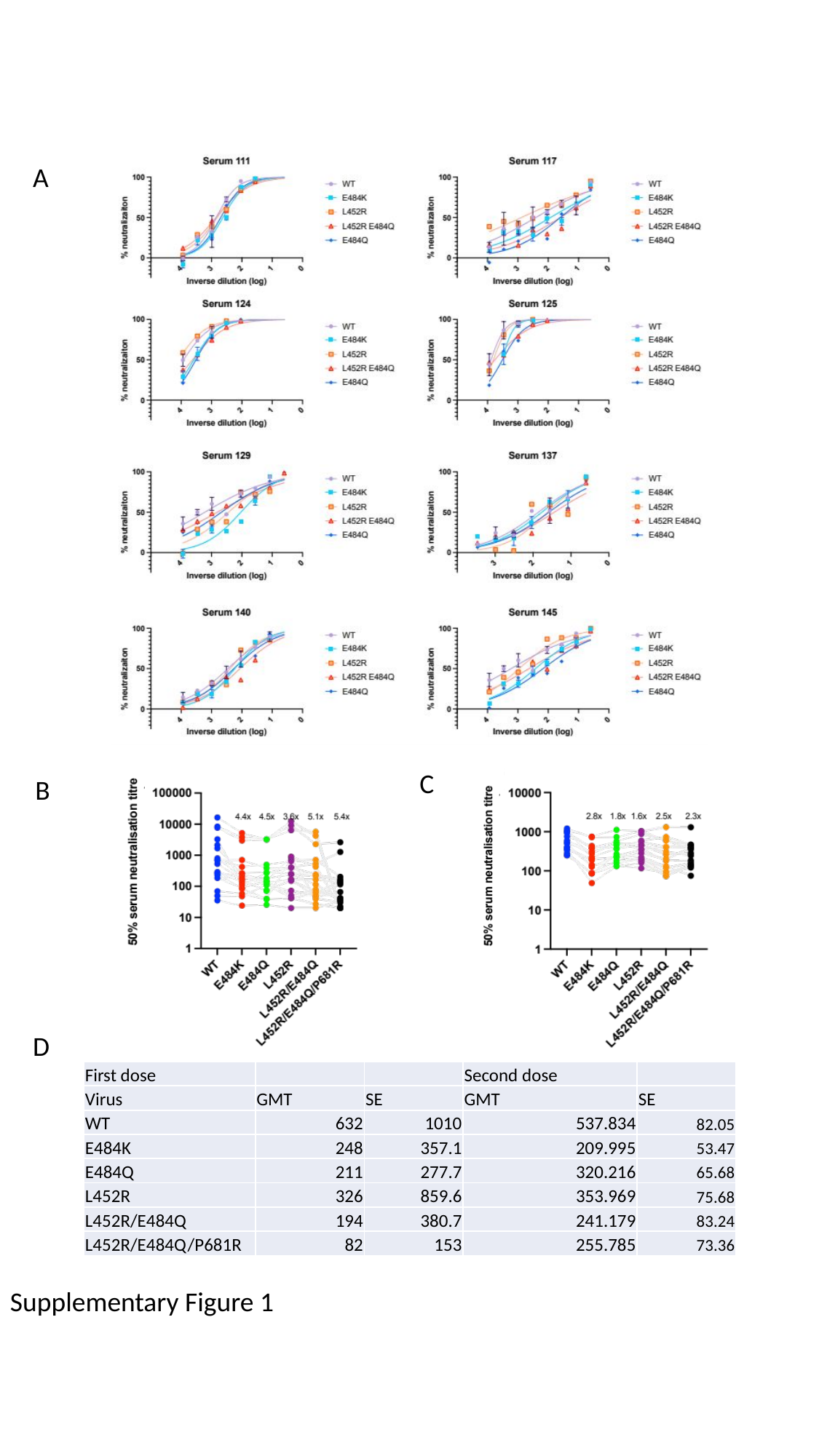

A
C
B
D
| First dose | | | Second dose | |
| --- | --- | --- | --- | --- |
| Virus | GMT | SE | GMT | SE |
| WT | 632 | 1010 | 537.834 | 82.05 |
| E484K | 248 | 357.1 | 209.995 | 53.47 |
| E484Q | 211 | 277.7 | 320.216 | 65.68 |
| L452R | 326 | 859.6 | 353.969 | 75.68 |
| L452R/E484Q | 194 | 380.7 | 241.179 | 83.24 |
| L452R/E484Q/P681R | 82 | 153 | 255.785 | 73.36 |
Supplementary Figure 1

## Slide 2
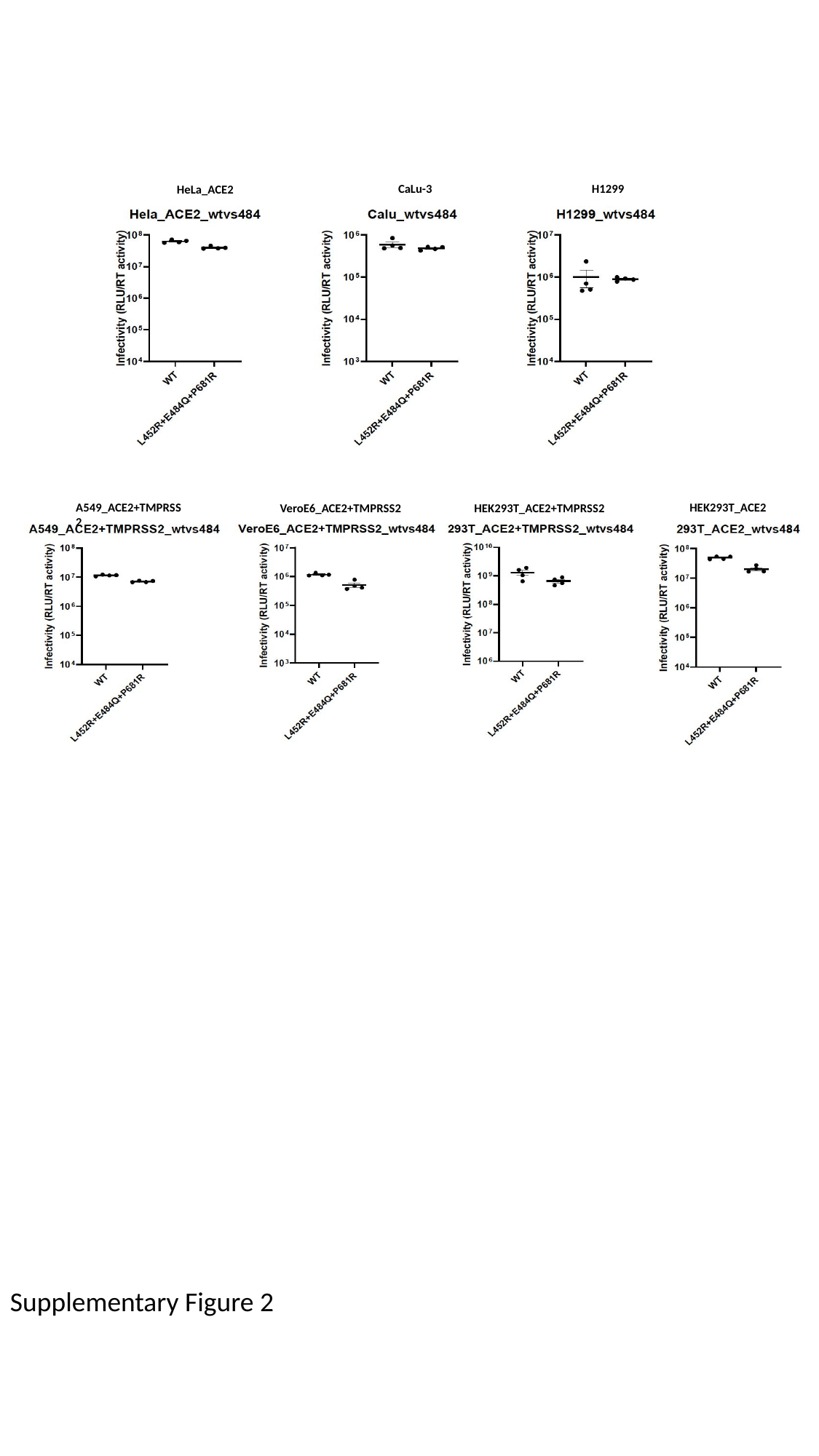

CaLu-3
H1299
HeLa_ACE2
HEK293T_ACE2
A549_ACE2+TMPRSS2
VeroE6_ACE2+TMPRSS2
HEK293T_ACE2+TMPRSS2
Supplementary Figure 2
